# Embodied collaborative writing in graduate dance education

**DOI:** 10.3389/fspor.2024.1330422

**Published:** 2024-03-28

**Authors:** Pirkko Markula, Janita Frantsi

**Affiliations:** Faculty of Kinesiology, Sport, and Recreation, University of Alberta, Edmonton, AB, Canada

**Keywords:** contemporary dance, embodied writing, new materialism, performance ethnography, dance course

## Abstract

This paper explores how embodied writing can inform teaching, learning, and research presentation in graduate-level dance education in a kinesiology faculty. The focus is on a graduate dance course “The Dancing Body in Motion”, which combines the anatomical analysis of the physical body, social theory, and lived dance experiences to promote more embodied and holistic teaching and learning. The authors, an instructor and a student of the course, share their experiences and reflections on the course through an embodied presentation of a dialogue that combines the instructor’s lecture notes, the student’s learning journal entries, and their reflections both separately and in conversation with each other. Their reflections offer insights into how the body and mind, material and social body, and practice and theory can all be brought together using embodied writing practices, such as a learning journal and performance ethnography, in a dance performance.

## Introduction

Dance training at the tertiary education level often prioritizes technique classes as a means to prepare the body for the physical challenges of a professional career. Although some theory or history courses may be included, they tend to be separate from the physical dance training. To reduce the gap between theory and practice, the current dance education literature calls for integrating more writing into dance programs throughout curricula (1, 2). In this context, embodied writing can explore the relationship between the mind and the dancing body to develop alternative ways for undergraduate dance students to practice reflection. This type of embodied writing can address the need for diverse and inclusive ways to educate dancers [e.g., (1)].

Somewhat departing from this approach to integrating embodied writing in undergraduate tertiary dance education, our work stems from graduate-level education in the Faculty of Kinesiology, Sport, and Recreation (KSR) where theoretical learning (the mind) dominates. In this context, embodied writing can assist in including the material body in research practice and writing. According to Bradshaw-Yerby (1), embodied writing “can enhance our creative fluency as writers, dancemakers, and ultimately, as interdisciplinary scholars” (p. 192). At the graduate level, dance technique, choreography, personal reflection, and research can all be used in embodied writing activities in a dance-based education setting (3). Harvey et al. (3) further emphasized that embodied writing can help overcome the body–mind dualism that places rational cognitive thought as epistemologically superior to embodied, experiential knowledge and, as such, disconnects research knowledge from the lived experiences of bodily and somatic ability.

In the sociocultural area of KSR, we focus on theoretically driven graduate-level education that enables the students to engage in critical and analytical thesis research. The students interested in studying dance to complete their degrees are also expected to employ critical thinking and a methodological approach to analysis in their research. However, the typical academic learning, research, and writing process can be enhanced by engagement in embodied ways of learning and writing that use the students’ lived experiences in dance as central aspects of their learning. In our view, researching dance as a social and cultural bodily practice requires multidisciplinary learning that utilizes embodied writing. Moreover, including embodied writing practices as a part of the dance students’ graduate education can increase the methodological and writing choices in their research [e.g., (4)].

In this paper, we, as a teacher and an independent dance artist and a previous student, highlight the possibilities for embodied writing and performance in a graduate-level dance course “The Dancing Body in Motion”, which we created specifically for Janita's degree in kinesiology with a focus on dance. In this collaborative and reflective paper, we reflect on how the course assignments based on lived body experiences can serve as sources for embodied research writing. Drawing on new materialism, we highlight how the dominant writing practices in graduate education can be informed by the performative dancing body. To enhance our research writing choices, we also employ embodied writing to present our reflections on the course.

## From embodiment to embodied writing

With an emphasis on the dancing body and scholarly writing in our course, we now highlight how the concept of embodiment can operate to bridge these two practices. According to Ulmer (4), embodiment has become an important topic in dance studies. Although commonly used, it has assumed multiple meanings [e.g., (4, 5)]. However, many dance scholars draw on Maurice Merleau-Ponty's phenomenological understanding of the moving body [see (4, 6, 7)].

In his “Phenomenology of Perception”, Merleau-Ponty (8) emphasized that, fundamentally, phenomenology provides a means for understanding how the living relationship of experience, grounded in perception, can help rediscover one's presence in the world. The physical body provides the mechanism for perception and, thus, is an essential element of phenomenological analysis as it enables knowing the meanings of the world. It is bodily action, the body's movement, that brings the meanings into being. Merleau-Ponty's phenomenology thus binds together the physiological body and the (psychological) consciousness of meaning-making. The body uses its senses to give expression to its movements that take expression as a basic intentionality toward the world. As such, the body acts as a mediator: it expresses our being in the world, and through it, we come to understand the world. Merleau-Ponty (8) used dance as an example: The habit of dancing is discovering the “formula” of movement that is recognized as dance. The dancing body “catches” and “comprehends” movement (p. 142–143), but also “grasps” its significance, and its figurative meaning as dance (7). Through certain behaviours and functions, such as dance, the body also gives new meaning to “meaning”. The moving body, thus, acts as the window to the world and united with consciousness, the mind, gives significance to what takes place around it. In the phenomenological framework, the individual, thus, is the starting point of understanding the world. In this sense, Merleau-Ponty's phenomenology is understood to move beyond the body–mind dualism—the separation of the physical body from the mind—that some dance scholars find prominent in westernized cultures (9, 10).

Centred around the body and its motility (the movement habits), Merleau-Ponty's approach is “well-suited in the in-depth portrayal of the corporeally grounded experience” of physical activity (11). As the world becomes meaningful through bodily action, phenomenology can illustrate the unique ability of the moving body and, consequently, how dance creates knowledge of the “being in the world”, thus justifying it as an essential phenomenon for human existence in the world. For example, the early work of Sheets-Johnstone (12) advocated a description of the “original, pristine body, the preobjective or preobjectivized body” (p. 133) of dance. To detect how the body mediates meaning-making, phenomenological analysis is based on a detailed description of how, by inhabiting the world, the dancer is open to experiencing and learning about the world. For this description to capture the world beyond perception, the researcher's own experiences should be bracketed out to ensure that the description pertains to the primordial world, not the researcher's reflection.

Merleau-Pontian dance studies continue to highlight the importance of “being-in-the-world” through the dancing body that, as a medium to the world, is open not only through its innate structures and general basic skills but also through cultural skills (such as dance skills) (13). Thus, examinations of perception and movement experience and how this solidifies into cultural movement in intersubjective relationships of the self to the others are central to the phenomenological descriptions of dance (10). Parviainen (14), for example, employed insights from several phenomenologists including Merleau-Ponty to distinguish between dance skill and dance knowledge generated through “an understanding of the subjective process whereby dancers understand, create, and use knowledge” (p. 22). More recently, Purser (15) who interviewed 16 professional contemporary dancers employed Merleau-Ponty's non-dualist conceptualization of “body-subjectivity” to suggest that their lived experiences allowed the dancers to be directly conscious of their bodies and “the here-and-now”. In her study, Rouhiainen (16) emphasized how intersubjectivity, the interaction with others in her dance space, enables a dancer to discover and respect individual differences, and thus, teach ethical relationships to others in the world. Other researchers have used the phenomenological approach to study the lived, embodied experiences of ballet dancers.

Aalten (17–19) argued that the material bodily practice and experience of ballet tend to be ignored by researchers on dance studies in favour of the readings regarding the textual material of dance. When tracing the embodied dance experiences of dancers in several ballet companies in the Netherlands, Aalten (19) observed that they generally treated their bodies instrumentally “as objects controlled by their minds” (p. 111). Aalten concluded that ballet, although intensely bodily, was also a strangely disembodied world of strict discipline that encouraged the dancers to view their bodies as objects of control only listened to when they did not perform to the required standards. The dancers knew about their bodies by “transcending” their pain, their shape, or their technical limitations. It was a body mastered by the dancer's mind. While Wainwright and Turner (20) also acknowledged the ballet body as an absent body prior to injury, they hoped to highlight the body, emotion, and identity as interconnected in their phenomenologically inspired approach. For them, embodiment encompassed the dancers’ physical, personal, social, and spiritual beings that emerged in this interconnection. When an injury prevented dancers from practicing their profession, it had a deep impact on their identities embodied through intense bodily training from a very young age. Both Aalten and Wainwright and Turner were outsiders gazing into a foreign ballet culture of hardiness and, as phenomenologists, did not include their own experiences in their studies. Generally, phenomenological researchers acknowledge that embodied experiences still need to be written to turn into knowledge [e.g., (5, 7)]. Parviainen (14), for example, acknowledged that “knowing in dancing always has something to do with verbal language; nevertheless, it essentially concerns the body's awareness and motility” (p. 13). Despite their focus on individual bodily experiences, the studies are written as traditional, realist research works.

Although phenomenology appears to provide an avenue to embodiedness, Ness (21) pointed out that dance phenomenologists, when bracketing out researcher's subjective influences to tap into true, untainted lived experiences, also tend to bracket out culture that also shapes dance and dancing bodies. To provide unique knowledge that is not accessible to observation, Ness continued that embodied practice should be able to reveal something that is produced specifically through dance in culture. Combining the embodied practice in dance with its cultural meaning turns into “embodied knowledge”: “a type of knowledge that arises from bodily practice of dance influenced by and informing cultural knowledge” (5). This understanding of embodiment moves beyond phenomenology to include multiple theoretical perspectives and the researchers’ own bodily experience of dance in its cultural context [e.g., (22–25)]. This understanding of embodiment emphasizes the researcher’s own dance involvement as a way of capturing the uniqueness of embodied dance knowledge more broadly. To make the embodied knowledge “visible” (23), it needs to be translated into words that then act as the entry points for a scholarly analysis. Despite including the researcher’s own experiences in the research text, the style of writing remains realist, academic writing.

To some dance researchers, embodiment as a concept that includes the descriptions of the body, the lived experiences of the body, and how these are culturally constructed has provided a tool to refocus their research writing. For example, in her writing, Browning (9) combined lyrical, personal narrative, and theory from literary studies and anthropology to highlight a body that emerged simultaneously as aesthetic, spiritual, political, and sexual both in her and the Afro-Brazilians’ everyday samba experiences. Savigliano (26) spoke “in bursts, splashes, and puddles” (p. 12) of fictionalized characters, metaphors, and historical events (de)constructed by her own decolonization process through the formation of tango. Barbour (27) stressed the importance of “literary techniques…to develop coherence, verisimilitude, and interest” (p. 102) in her autoethnographic “embodied knowing” (p. 101). Because of the uniqueness of embodied knowledge, translating it into writing tends not to be a straightforward process [e.g., (9, 23)]. To explore the connections between embodied dance experience and writing, some researchers have turned to “embodied writing” that further captures the moments of lived bodily experience in the social and cultural contexts.

### Embodied writing

Cooper (2) described embodied dance writing as “focused, intentional, and full-bodied descriptive writing that is inclusive of an array of sensory mechanisms and explores all types of dance practice” (p. 54). This type of writing reveals the lived body experience in dance as portrayed in words. As Bradshaw-Yerby (1) elaborated: “Embodied writing is the practice of *in-body* writing. It is a sweaty, dynamic, limbs-flying-through-space kind of writing practice” (p. 193, emphasis original). As such, embodied writing creates opportunities to “invite habits of dancing” into writing (1). Ulmer (4) explored how dance, as an embodied choreographic practice, can change writing. As a form of embodied writing, her choreographic writing acts “as visual text in which words move, pause, gain emphasis, and flow as if dancing across the open page” (p. 25) to openly challenge the realist research writing tradition. When writing dance as embodied methodology, the researcher is to “experiment with different ideas of what it means to write dance” (p. 41). Using three different perspectives, phenomenology, new materialism, and Gilles Deleuze's poststructuralism, Ulmer explored “how the written text can be re-written, re-visualized, and re-imagined” to give opportunities for embodied writing (p. 45). Our approach to embodied writing practice in graduate dance education draws also specifically from new materialism.

New materialism is a heterogenous approach to social sciences and humanities research that draws from multiple theoretical and methodological traditions [e.g., (28–31)]. Although scholars from diverse disciplines engage and interpret new materialism in a variety of ways, their approaches share several common features. In our work, we draw from first, the new materialist call to reduce dichotomous thinking in the humanities and social sciences and second, the need to challenge the current onto-epistemologies that deepen divisive dualisms in research.

Similar to dance studies, new materialists find “dualism” that separates the thinking mind from the practicing body to dominate much (social) science and humanities thought [e.g., (30)]. Scholarship based on such dichotomies, according to new materialists, inadequately captures the complexity of the world when it places the “matter” and materiality (i.e., the body, nature, non-human objects, environment) as separate from humans, their ideas, values, politics, and society [e.g., (32)]. Consequently, social analysis or scientific experiments alone are insufficient if we aim to understand how “things” around us happen in everyday life. For us as sociocultural researchers of dance, this indicates that mere social analysis is “inadequate for thinking about matter, materiality, and politics in ways that do justice to the contemporary context of biopolitics and global political economy” (28). We now need to reconsider the division between science and social science, nature and culture, matter and social, body and mind, or practice and theory that have been the organizing principles of much modern tertiary education and research that derive from the dualist world view. In our case, a turn to new materialism provided a chance to experiment with different approaches to graduate dance education to problematize the natural science–social science, mind–body, and theory–practice divisions in kinesiology. This inspired the philosophical premise of the “Dancing Body in Motion” course that drew attention to how the material interacts with the social in the world of dance.

### New materialism and embodied writing

Similar to Ulmer (4), the new materialist approach expanded opportunities to learn and write about the embodied experiences of the dancing body. Although new materialists advocate for both natural and social science knowledge, they are critical of the current research ontologies that offer only limiting ways to view phenomena in the world (28–30). The realist ontology of natural sciences, the new materialists argue, treats bodies merely as passive objects of which functions and mechanics are to be objectively observed by a scientist without their influence on the research process [e.g., (33)]. Following new materialist Barad (33), the phenomenon under investigation is inseparable from the scientist who creates the measuring instruments and conducts the measurements that then impact the scientist's observations. In this view, the observations of the biological body are constructs similar to the social observations of it (34). In our course, we did not conduct scientific experiments regarding, for example, the forces affecting the dancing body, but we chose to engage with anatomical and biomechanical knowledge that was presented in its own section. However, instead of discussing published, realist, scientific biomechanical research on dance or using scientific writing style in the course assignments, we aimed to move beyond the quantitative, objective research by engaging in practical, embodied learning of how our dancing bodies work as biological entities. We used qualitative movement analysis to consider, experientially, how to practice dance techniques in anatomically safe ways. Following the new materialist premise, however, this biological body still interacts with the social world of dance.

To consider the social meanings, we studied a number of social theories and their understandings of the dancing body. Through an analysis of the main social science concepts in dance studies, we also aimed to problematize each theory to give Janita the choice of engaging with a theoretical perspective meaningful to her project on the dancing body in motion. However, social sciences and humanities researchers represent their observations through language that privileges the human who makes meanings of the world but leaves the material world without agency (28–31). Therefore, detecting the social meanings alone will not provide a holistic picture of, for example, the dancing body. As Wainwright and Turner (20) argued, both “biological reductionism” and sociological “deconstruction” are insufficient for understanding dance. This new materialist premise provided ways to represent embodied knowledge in alternative ways to a research paper in the course. This new materialist premise set the stage for the “Dancing Body in Motion” course and also for our exploration of embodiment and embodied writing. We now discuss the course content and the writing practices embedded in it in more detail.

## The “Dancing Body in Motion” and embodied writing

Although initiated by physical educators (35, 36), many postsecondary level dance programs in North American tertiary education have migrated to the Fine Arts faculties (37–39). Our Faculty of Kinesiology, Sport, and Recreation (KSR), previously named as Physical Education and Recreation located within a large university with a strong research orientation, has retained both undergraduate-level dance courses and graduate-level dance education. The faculty also offers several graduate degrees (e.g., MA, MSci, MCoach, Ph.D.), and it is possible to choose dance as the primary topic for one's graduate research. As a result, several graduate students focus on dance in their MA or Ph.D. studies although there is no graduate program in dance *per se* [see (40)]. The “Dancing Body in Motion” course was created as “a directed study” specifically as a part of Janita's MA studies in the KSR. Pirkko as the instructor and Janita as the student collaborated to create its contents. Following a new materialist premise, the course focused on understanding the dancing body simultaneously as a material and social body. Consequently, the learning objectives included understanding the basic muscular–skeletal functions of the dancing body; the evaluation of the meanings of the dancing body through phenomenological and critical perspectives; and the representation of dance research through performance ethnography informed by new materialism and Gilles Deleuze's poststructuralist theory.

Based on the learning objectives, the course was divided into three main parts: movement analysis in a studio space that used anatomical and biomechanical concepts to examine functional dance technique through qualitative dance movement analysis supplemented by material from dance kinesiology and anatomy texts; a social theory section in a lecture room that focused on phenomenological discussion of embodiment and critical analyses of the dancing body constructed in its social context with scholarly articles as reading material;^1^ and a performance ethnography section, in a multipurpose studio space, drawing on poststructuralism to combine new materialist principles with movement practice.

To consider the interaction between the material and the social dancing body and the lived embodied learning experiences, the course included both practical and theoretical assignments that involved personal reflection. The three assignments were as follows: practical movement analysis with a focus on designing a dance exercise for a specific part of the body; a learning journal comprised of observations and reflections on the course content from the duration of the course; and a performance ethnography to draw together the three aspects of the course. In this paper, we focus on two of the assignments—the learning journal and performance ethnography—that highlighted the dancing embodiment: the physical body, lived experiences of the body, and the cultural construction of the performing body.

### Embodied writing: the learning journal

In our course, the learning journal aimed to capture embodied writing that reveals the lived body experience in dance as portrayed in words (2). This type of embodied writing was to create opportunities for Janita to “invite habits of dancing” into her writing (1). The learning journal comprised observations and reflections on the course content from the duration of the course. As the exact format of the learning journal was open to the student's organization, Janita reflected on her learning against a variety of issues in dance, such as common dance practices, different dance forms, and her personal, lived experiences as a student and instructor of dance, and, overall, her identity as a dancer. As it encouraged critical discussion and analysis of Janita's own experiences in dance, common social norms, expectations, and the dancing body ideals, the format of the journal allowed for vulnerability, sharing of negative experiences, and analyzing and understanding them.

### Embodied writing: performance ethnography

In the course, embodied writing served also as a way of experimenting with alternatives to usual writing assignments that at the graduate level typically consist of writing a research paper. According to Aalten (19), the most meaningful dance moments are the ones where “the mind of control” and “body in performance” unite. Consequently, our course culminated in a performance ethnography that aimed to join the elements from the qualitative movement analysis of the material dancing body, the sociocultural meanings of the dancing body, and the performative ways of representing new materialist-inspired work that made this embodied knowledge “visible” (23) through both words and bodily movement.

While a term initially coined by Denzin (41), the idea of ethnography as performance (rather than a written work) has evolved in several directions. Harrop (42), for example, distinguished between three interrelated trends in the ethnography of performance: an embodied, affective, and sensory ethnography that privileges the encounters between the ethnographer, participants, and practices as key to understanding and knowledge; ethnographic practice amidst the migration, diffusion, revival, appropriation, and commodification of performance; and the interface of academic disciplines with the idea of performance. Denzin's original performance ethnography can be seen as the foundation for the latter trend.

Pirkko's work (43, 44) has drawn on Denzin's (41) characterization of performance ethnography as a sociopolitical act of intervention, a form of criticism that uses aesthetics to locate bodies, individuals, and/or events within the intersections of institutional and cultural politics and embodied experiences. For Denzin, it is a way of “bringing culture and individual into play” (p. 9) through social science that resembles a performance to then become a sociopolitical act that “can contribute to radical social change, to economic justice, to a cultural politics” (p. 3). As inherently political, performative social science “puts culture into motion. It examines, narrates, and performs the complex ways in which persons experience themselves within the shifting ethnoscapes of today's global world economy” (p. 8). The researcher is an integral part of a performance social science research project putting the research into motion to change the social reality. As such, performance ethnography must combine artistic excellence with “a moral responsibility…to produce an active intervention” (p. 18). Adding to calls for embodied writing in dance, Denzin's (41) performance ethnography moves beyond “traditional” social science research texts by employing live performances to advocate social science research act as “a form of kinesis, of motion, a decentering of agency and person through movement, disruption, action” (p. 10). In this sense, it serves as a new materialist research practice that brings together the material body and social science into a performance that puts culture, holistically, in motion. In our course, Denzin's (41) performance ethnography provided a framework to “contribute to an epistemological and political pluralism that challenges existing ways of knowing and representing the world” (p. 8). While Denzin did not limit the theoretical or artistic options for performance ethnography representation, Pirkko's work has focused on the body's doing and its force, informed by a poststructuralist, Deleuzian reading. The performance ethnography section in the “Dancing Body in Motion” was also informed by a poststructuralist premise of the doing, physical body making visible social science concepts to put the research culture in kinesiology in motion.

The performance ethnography assignment as a way to represent dance research combined theoretical content, personal experience from Janita's learning journal, and anatomical knowledge with movement expression, and as such, it drew together all aspects of the course. It also encouraged the use of spoken and written texts as a part of the performance to include both the theoretical concepts (the mind) and dance practice (body) in the performance. It was the embodied experimentation of alternative ways of representing scholarly writing that made the new materialist premise of moving beyond body–mind dualism visible to understand the world both as material and social through dance. Despite her significant performance experience in contemporary dance, Janita, at the time, had no previous experience of performance ethnography as a research representation. Pirkko presented one of her previous performance ethnographies in class to demonstrate how dance movement can enhance academic theoretical discussion. We then began creating the performance ethnography collectively.

Based on Denzin's (41) premise of performativity that he defined to include the researcher's experiences; to disrupt “discourse” and theory; and to include the material (dancing) body, Janita was, first, to take three memorable personal experiences from her learning journal and, second, a theoretical concept from the lectures that was connected to the experiences. Drawing on her personal experiences and reflections as a Pilates instructor, dance teacher, and a dancer, Janita's choice of concept was dancer's self-construction. She then started experimenting with what it meant to move inspired by her personal experiences, such as the struggles of not feeling good enough, dancing through pain, but also the feelings of happiness when dancing. To further contextualize dancer's self-construction, she included elements of the broader theoretical perspectives discussed in the course: phenomenology to discuss dancers’ experiences, critical theory to examine different dancing identities, and poststructuralism to question the dominant identity categories. From the initial movement experimentation, Janita built in more theoretical text chosen to support the original (or discarded) concept of self-construction; further references to her struggles with functional dance technique; and more movement themes. We developed Janita's performance ethnography section by section (first by adding movement material to her personal experiences and then including theory and more movement themes) with Pirkko providing continual feedback during the creation process.

To further build her performance ethnography, Janita explored the combination of movement and text, how to use space and tempo, and how to create movements that elaborated on the themes of the texts. Drawing on contemporary dance techniques, she created abstract movements alongside the words and ideas of the texts. Sometimes, she presented the movements and text simultaneously; sometimes, she separated the sections into movement or text only. In addition, Janita used sheets of paper as props to read and elaborate on the theoretical discussions, her breath, and some percussive movements to give her movement tempo. After one week of intensive work, Janita was ready to show a preliminary version of her performance ethnography in a feedback session. The final version was performed the following week. The performance ethnography assignment also included writing an eight-page research paper due after the performance.

The evaluation of the performance focused on such aspects as appropriate theoretical content, comprehension of theory, performance of text, and effective use of personal experience. The practical movement part of the performance ethnography highlighted the representation of text through the body and was evaluated through the use of space and time, the dynamism of movement quality, choreographic effectiveness, and performance quality. Through the combined theoretical discussion and dance, the performance ethnography created a seamless interaction between theory and practice, mind, and body. This embodied experimentation made the new materialist premise of moving beyond body–mind dualism visible to understand the world both as material and social through dance.

### Embodied research writing

As discussed earlier, embodied dance experiences have served as sources for (alternative) writing practice for some scholars (1, 4, 9, 26, 27). Ulmer (4), for example, discovered that embodied writing, with its emphasis on experience and perception, turns into a “choreographic writing” that flows, stops, accelerates, and has silences in a similar way to a dance phrase. The coherence and flow of the phrase emerge through the visual movement of the text rather than the use of conventional tools of realist research writing. This type of writing also challenges the conventional uses of space on a page and, thus, will necessarily look different on the page.

**Figure 1:**
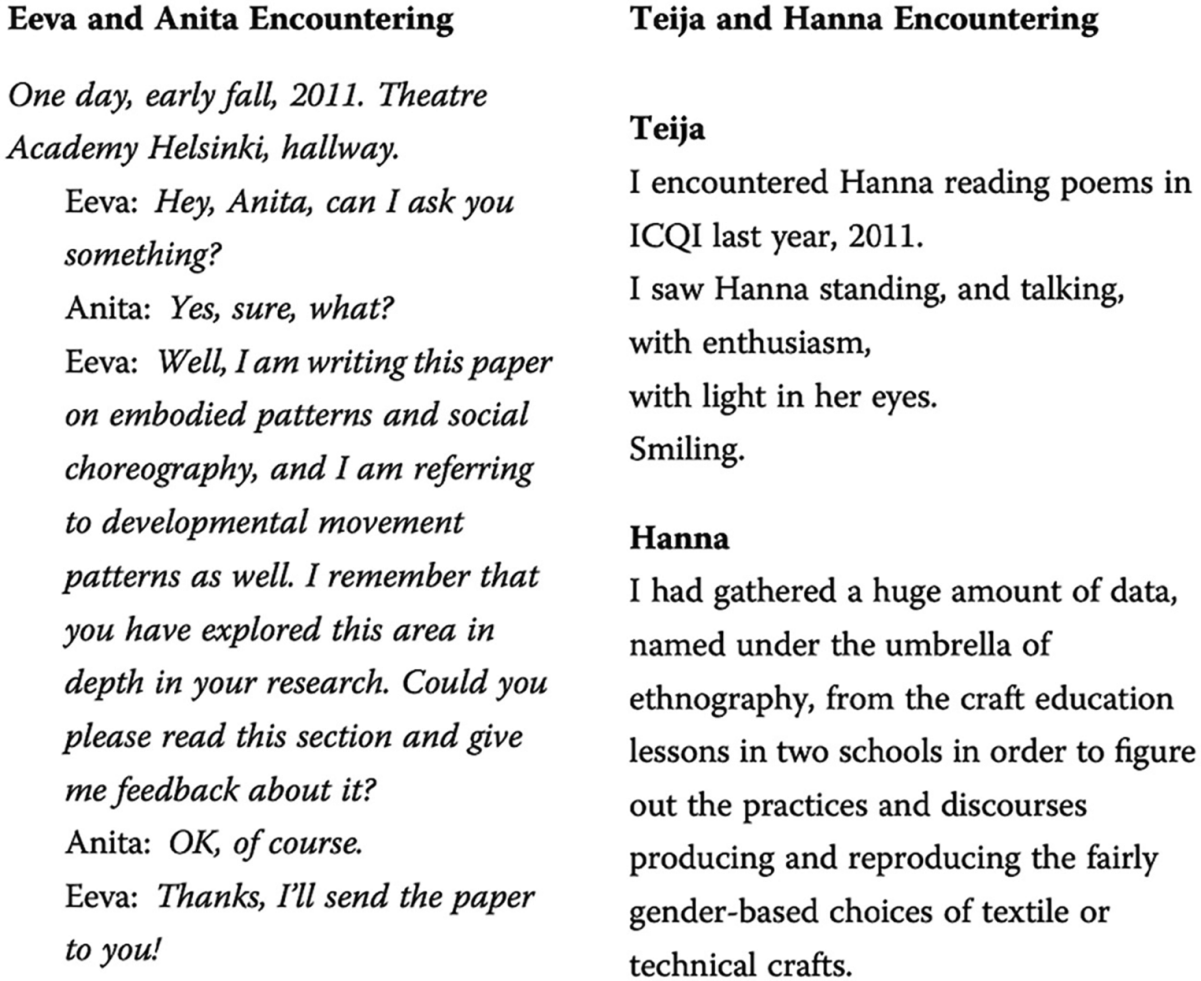
An embodied presentation (45).

Löytönen et al. (45) advocated embodied writing as a way to engage theoretical perspectives with artistic writing practices. Their collaborative embodied writing explored their “newfound awareness of our ways of be(com)ing writing (and presenting) academics in our (always already) embodied and moving lives” (p. 236). Inspired by Deleuze (46), they approached themselves as “thinking, feeling, and writing academics through playing (and fumbling) with patterns and possibilities in collaborative and embodied writing and presenting practices within academia” (p. 236). Through the “zigzagging” movement of their writings, they engaged both embodied and literary encounters in their lives into a text that simultaneously played their “multiple experiences, accounts, and stories that fold in and back on one another and rippling into” scholarly and artistic practices (p. 236). Their writing was designed to disrupt the comfort of taken-for-granted academic reading and writing that relies on coherent structure with clear sign posting. Instead, the reader was challenged to make connections between the authors’ stories through the use of different fonts, text formats, dialogue, and spacing on the text on the page. On the page, their writing, thus, appeared quite different from the usual scholarly research text (Figure 1).

We were also inspired to experiment with this type of embodied research writing. When we reflected upon our experiences—which were both material and conceptual, visceral and thought out, bodily and mindful, dynamic and still, and individual and cultural—instead of academic, realist, scholarly writing, we chose to experiment with a form of embodied writing that flows through different phrases based on our collaborative reflections of the “Dancing Body in Motion” course. Similar to Ulmer's (4) choreographic writing, we used different ways of spacing the text on the page, italics, and bolding to make the writing move with our experiences of the dancing body in the course. Following Löytönen et al. (45), we employed a first-person dialogue to disrupt the conventional model of academic writing and draw the focus on the dancing body as simultaneously material and social.

### Empirical material for embodied research writing

We drew from several sources of empirical material to create our embodied writing presentation. We returned to the course materials (syllabus, lecture notes, detailed descriptions of the assignments) to collaboratively reflect on the actual flow of the course. As a result, we have included some of the PowerPoint slides from the lectures to add further visual flow to our embodied writing presentation. Janita carefully re-read her learning journal entries (11 entries, 2–5 pages each), some of which we have included in the embodied writing presentation in italics. In addition, we independently wrote our reflections on four topics that we agreed upon at the beginning of our collaborative writing process of this paper: general impressions of the course, movement analysis section of the course, learning journal as an embodied assignment, and performance ethnography as an embodied assignment. Janita wrote additional reflections regarding the meaning of the course in her current career as a professional artist. Each of these reflections was three to four pages long. Finally, we viewed two videotaped recordings of Janita's performance ethnography together followed by a discussion of each performance. The dialogues that we had when collaboratively writing this paper are centred on the pages of our embodied writing presentation. Our embodied research writing practice then acted as an analysis of the empirical material that found its flow during the writing process. We obtained the ethical approval for our work from the University of Alberta Ethics Board.

## Embodied presentation: reflections on embodied writing in the “dancing body in motion”

Janita:

To begin, I acknowledge that dance is a physical activity; thus, the body is central to it. However, an extensive emphasis on the body puts pressure on how we encounter different bodies.

Pirkko:

As a sociocultural scholar of physical activity, I embrace theory. It enables a researcher to describe and critique phenomena, but also opens up avenues for thinking differently and then creating social change. But does the emphasis on the foundations of theory move us away from the many cultural issues that dancers face every day? How do we not separate theory from dance practice and lived experiences to fully embrace its embodiment?

Janita:

Social theory is rarely combined into dance education. During my undergraduate studies in sports sciences, I was excited to discuss the sociocultural issues of dance in particular.

(Too) Often in a dance class, we focus purely on the physical side of dance. When I was studying dance in Finland, the subjects included dance technique, choreography, and cultural history. There was no discussion about the social side of dance and the problematic issues, such as eating disorders, comparison, and pressure.

Why do we ignore these issues? Are they too difficult for us to bear?

Pirkko:

As a contemporary dancer, I move. Having experimented with multiple ways to include embodiment—combining social theory and practice, sociocultural concepts and the moving body, writing, and dancing in my research—I found new materialism. I now wanted to translate new materialism to inform my teaching and was excited to design a directed study for my graduate student who could be potentially interested in knowing both the material and social dancing body and alternative ways of embodying them in a performance.

Janita and I had similar backgrounds in kinesiology, and, thus, I knew that Janita would embrace a discussion of including the physical aspects through anatomical and biomechanical issues of the dancing body and possibly be interested in safe dance technique having experienced a dance injury herself. At the same time, Janita had to write a thesis to complete her sociocultural studies of physical activity. Therefore, it was necessary to introduce social theory to the mix for a more complete embodied understanding of lived dance experiences. I also knew that Janita, as a dancer, would take on an opportunity to perform, but that the performance, from a new materialist research perspective, needed to go beyond physical to explicitly include theoretical concepts, text, and social research into an embodied presentation. The idea for the “Dancing Body in Motion” course was born.

I was not assigned to teach this course as my regular teaching load typically consists of faculty-wide compulsory courses on research methods, social theory, or Ph.D. research. As an additional self-selected choice, I had a chance to disrupt the reliance on theoretical (mind) lecture-type graduate teaching characterizing our faculty: I scheduled the course as three intensive workshops, each over 3 days; I booked a variety of class spaces from a studio to lecture room to multipurpose room; and I scheduled a separate performance space at the end. This, I hoped, enabled us to engage in the more embodied practice of learning and presenting the dancing body in its sociocultural context.

### Embodied ways of learning about dance: the material body

Janita:

In dance, the work to improve the body is endless; we always work towards something more. When can we be happy about ourselves, our bodies, our technique, and our dancing—or can we ever?

Because of my background in sports sciences and as a Pilates and fitness instructor, I have always been interested in human anatomy. I was excited about the practical movement analysis, as it allowed me to focus on the body from the natural science perspective, which felt familiar from my previous studies. As a graduate student in the sociocultural studies of dance and physical activity, completing a practical movement analysis was also different from my other courses.

Pirkko:

The first section of the course stemmed from my own experiences of being assigned to teach biomechanics in a kinesiology department. Although a daunting and frustrating experience initially for a non-biomechanist, I still embrace the insights into how the body moves anatomically and how this knowledge can inform safe dance techniques. As a kinesiology student, I, of course, had to take several courses in anatomy and biomechanics in which I, with varied success, learned the muscles, the correct equations to calculate angles, speeds, velocities, accelerations, forces, the involved work and power, momentum and energy to understand the mechanics of movement. However, I did not learn to move in the courses. Therefore, I wanted to teach anatomical and biomechanical knowledge in a more embodied way: to be applied to dance movement considering the cultural and aesthetic requirements of dance techniques that can exploit the extreme capacities of the body.

Janita:

As dance education typically focuses on dance technique and the stylistic characteristics of a dance style, anatomy and the muscular–skeletal functions of the body are often overlooked. However, understanding how the body works contributes to proper alignment, safer use of the body, and injury prevention. While discussing and demonstrating the safe, anatomical execution of dance techniques, we also questioned why certain exercises are performed. This brought in the cultural connection to dance training in a practiced, embodied way. Therefore, the qualitative movement analysis allowed me to tune into the dance as an embodied practice: refine common technique exercises, how we execute them, why we perform them, and how they feel in the body, instead of simply copying or continuing to teach a movement without knowing how or why it is performed.

Not everything in dance is natural or safe regarding how our body functions. Engaging in a careful analysis of an exercise forces one to question and problematize the meaningfulness of the movement, which is important since plenty of dance exercises are taken for granted and left unquestioned.

**Pirkko:** Was the movement analysis useful to you? How do you use all that knowledge now, or do you use it?

**Janita:** I totally use it! In two ways: First, injury prevention in my own training, to know the anatomy of the body when doing certain exercises. We do not always talk a lot about it during classes when it is more about learning the sequences. Second, when choosing my exercises for teaching.

Pirkko:

As I could not find a suitable course book for my approach to dance movement analysis, I used handouts and photocopies from different dance kinesiology texts and anatomy books. I am not sure if the reading materials delivered this way were the most helpful. I am not sure why I wanted to proceed body part by body part—I am not sure if it worked to divide the body into parts. In hindsight, I would now try to experiment further with how to use the anatomical knowledge to understand how the body works together more holistically in the embodied context of dance. My impression now is that this section was somewhat distorted, and although profoundly important and interesting to me, I was not able to teach optimally to facilitate Janita's learning of the material bodies as central to the embodied practice of dance.

Janita:

During our movement analysis weekend, we discussed the use and meaningfulness of external rotation of the hip (turnout). Based on our discussion, this common western concert dance function broadens the range of motion, offering more movement possibilities. In some everyday life movements (e.g., certain kinds of turns), turnout occurs inevitably. Since our hip joint is capable of rotation, why not practice it regularly? At the same time, however, I acknowledge that the constant use of maximal turnout, as it exists in ballet for example, does not appear in everyday life situations. It is quite an unusual and unnatural position for a human body. Therefore, turnout is not all good or bad, but it can become problematic when forced as an essential in dance.

Suddenly, I was meticulously aware of all the joints and muscles as well as the functioning of my body.

Because we do not usually discuss the exercises this carefully in a regular dance technique class, this section of the course made me temporarily hyperaware of the physical body.

Pirkko:

As the entire course was delivered as a series of intensive workshops (4 h each day), to more fully engage with dance embodiment, I aimed also to engage Janita with personal reflections and concerns at the beginning of each class before embarking on the more conceptual issues.

Janita:

While dance had not always made me feel great about myself, I was hesitant to share the negative experiences because I still loved to dance. However, starting the course by sitting in a circle and sharing our honest (also negative) experiences in dance set the tone for personal reflection in the learning journal. Without our discussions, it would have been easy to consider my experiences purely individual, but framing them through the sociocultural lens helped me understand how they reflected the social norms of how to be a dancer. We need to share our experiences and consider where they stem from to question and challenge them and create change in the dance world.

Sometimes my physical limitations and technical “flaws” (they certainly are not flaws but can be rendered as such by the dominant ideals of a dancer body) make me feel less of a dancer.

**Pirkko:** It was interesting how the practical movement analysis inspired the discussion about body image. Although I wanted to challenge you to consider dance technique as a cultural construction rather than purely physical, it was not my intention to relate anatomy to the dancing body ideals and body image at that point.

**Janita:** But it makes sense that the anatomy discussions brought on the body image topic as well. The body plays such a big role in dance, and when we talk about the technique, we often start to think about the cultural ideals too.

**Pirkko:** It was quite powerful how you then questioned if you even are a dancer in your learning journal.

**Janita:** Right, that came up multiple times in the journal.

Pirkko:

I still remember so clearly how the discussion turned into our histories as dancers: how each of us had some (or more) bodily deficiencies that marked us as not promising dancers; how we had heard that from our teachers, sensed it in the technique classes, and felt demoralized and failed. While we now had come to terms with these events in one way or another and created our own niches or unique ways of continuing to dance, the similarity of our past experiences was tangible. Perhaps, we became scholars due to these embodied experiences—to critically analyse the events, our reactions, and possibilities of transforming the dance culture.

Janita:

The negative issues in dance, pain, injuries, eating disorders, and neglecting the body, to name a few, are not shockingly new but somehow easy to put aside. Even though these issues are on the table, little to no change occurs, and we still keep on dancing. Perhaps dance is such a significant passion for us that we do not want to believe these issues; bear the pain that dancing may not be good for us. Or perhaps it is the firm belief that “if you want to be good, you have to suffer”. (19)

### Embodied ways of learning about dance: the socially constructed body

Janita:

How can it be that something that precious to us can also make us feel bad about ourselves? These reflections have made me turn to an academic exploration of the self in dance.

As a form of physical activity, dance highlights the material body, which makes movement analysis important. However, only focusing on the physical side contributes to the separation between the body and mind, and natural science and social theory. Combining this knowledge, using theory to reflect on lived experiences and connecting the material body with the social body contribute to a better embodied understanding of the dancing body. The material dancing body doesn't exist in a vacuum; it exists within its sociocultural context.

Pirkko:

As a dancer/researcher, I believed that it was necessary to add a section on social theory to further understand how the dancing body is socially constructed and then to break out from the limitations of social construction. Although we did not have time for a full-length social theory course, I chose to introduce two strands of social theory, phenomenology and critical (feminist) theory. Aided by articles from research journals (12, 16, 19, 47), the phenomenology section focused on highlighting embodied ways of knowing and knowing the dancing self through concepts of perception, the self, and intersubjectivity.

Janita:

Stemming from the feelings of inadequacy, I often question who I am as a dancer or whether I am a dancer at all. Phenomenology, however, makes my experiences feel valid. Sheet-Johnstone (12) notes that there are “many ways of being a body in dance” (p. 133). Acknowledging the multiple ways to experience dance reassures me—my experiences are just as valuable and sufficient as anyone else's.

**Figure 2:**
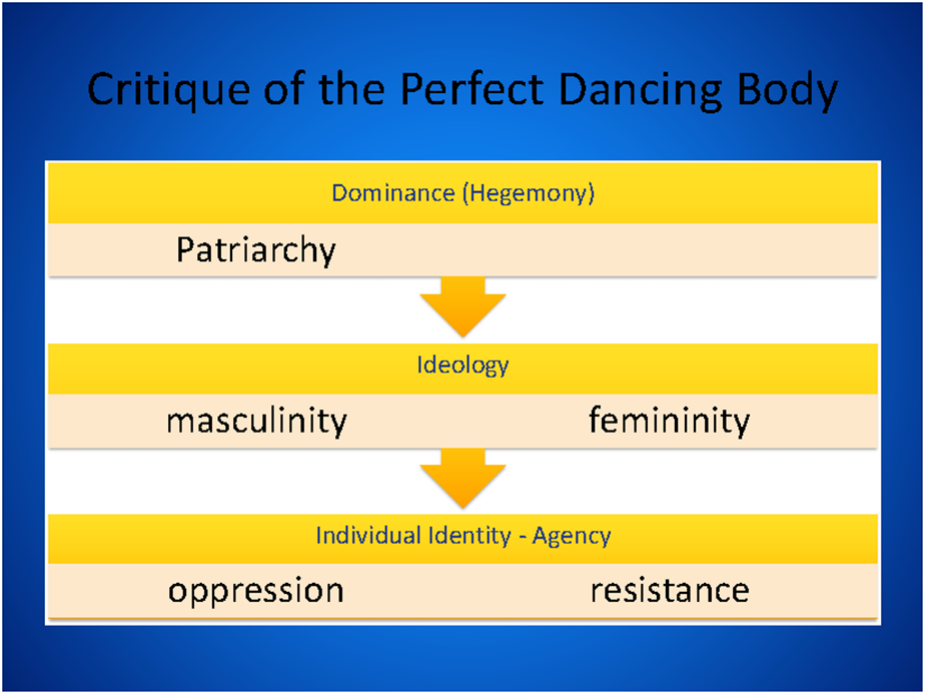
A slice from lecture 'dance and identity', The dancing body in motion.

Pirkko:

The critical theory part introduced identity-based politics in dance through dancers’ experiences of pain, injury, and body image as well as identity-based differences in the body politics of dance through critical feminist theory concepts of hegemony, ideology, and agency [e.g., (48)] (Figure 2).

Janita:

The pressures of the dancing body ideals were obvious in my learning journal. I connected the physical standards to the social body and discussed how I defined myself as a dancer through them. Combining social theory and natural science knowledge was valuable, as it provided a deeper embodied understanding of where the ideals and practices stem from and how they have become social norms in the dance world.

Why do I sacrifice my movement technique and safety for a higher grand battement or greater turnout? I feel pressured to do it to be a good dancer, a “real” dancer. I acknowledge that greater range of motion can offer more options for performing, but I am questioning the dominant ideals that determine how a dancer should act and look.

Pirkko:

Although we sat in a class room, I aimed to connect the theoretical concepts closely to dancers’ everyday lives, particularly the issues of cultural expectations of the feminine dancing body, to encourage embodied understanding of dance. This was particularly urgent as I recollected our earlier discussion on the “deficient” dancing body and its impact on a dancer's self and career.

Janita:

We discussed some bodily concepts, such as pain, through a social theory lens. In my learning journal, I reflected on how the lack of body awareness terrified me, yet I still witnessed that in my group fitness participants and myself. I connected a recent injury I experienced to the course materials and the social pressure to push the body.

Last term, I tore my hamstring in a dance class, and I am honestly not surprised by the injury. Before the class started, I felt that my mind and body were not “quite there,” not present for the class. I still started moving like nothing was wrong in my body—the thought of slowing down did not even cross my mind.

Pirkko:

This aspect of the course was the most familiar territory to me as I habitually teach social theory to kinesiology graduate students on sport, exercise, and health. The sole focus on dance, however, was a pleasure: there was a close connection to embodiment as my personal dance experiences intertwined with my identity as an academic researcher.

Janita:

Learning about different theoretical approaches felt meaningful when reflecting on past experiences. They validated my experiences, offered explanations for my feelings, and helped me understand that the experiences I thought I was not alone with were actually common in dance.

Pirkko:

The purpose here was also to further guide Janita toward her thesis research project that needed a strong theoretical framing.

**Pirkko:** You shared some reflections from different theories, like phenomenology, in your learning journal. Are you still using those theories in your work?

**Janita:** That's a good question. Poststructuralism, which I used in my thesis, guides my teaching and artistic practice. But the others… I guess I do use the phenomenological perspective when discussing and understanding experiences, the embodiment. But then critical theory…

**Pirkko:** Critical theory is often used related to equity, diversity, and inclusion. You probably use it in your Dance 200 “The Spectrum of Dance in Society” course that you teach at KSR when you discuss issues of representation.

**Janita:** That's right. It did inform a lot of the planning of Dance 200 and how I included different dance styles and showcased dancers from different cultures, races, and abilities, for example. So, critical theory does also inform the work that I do now.

### Embodied presentation: the matter and the mattering of the dancing body

Janita:

Moving helped me understand, discuss, and clarify theory when creating my performance ethnography. Perhaps my being a kinesthetic learner made the theory feel more comprehensible when explored through movement. I am not sure if using movements in the learning process works for everyone, but it is still valuable to give different ways of exploring and learning a chance to find what works best for each individual.

Pirkko:

Although I had created several performance ethnographies to represent my research, I had never taught how to do it to anyone and thus, using performance ethnography as a class assignment was an exciting new, but challenging opportunity for me.

Upon reflection, I did not have a clear idea of how I had created my own performance ethnographies. There was no analysis technique to use to integrate a conceptual framework into empirical material and then to research representation in a manner that is detailed for most qualitative methods. Therefore, I began this section of the course with a brief explanation of Denzin's (41) performance ethnography, and then, finally, I revealed how my idea of new materialism guided the entire course design. The performance ethnography was now to embody a new materialist approach by being not only a dance performance but by embracing performativity that Denzin defined as:

- Including the researcher's experiences (learning journal)
- Disrupting “discourse” (social theory)
- Including the material body (movement, dance)

Following Denzin, our performance ethnography work was to move in the space between

- Doing: performing
- Done: the text

to turn into performative social science. The new materialist approach guided the performance ethnographer to write with theory to think differently about bodies through different representation practices from realist, scholarly writing. As a new materialist researcher, I asked Janita: How does matter make itself felt through embodied practices?

**Figure 3:**
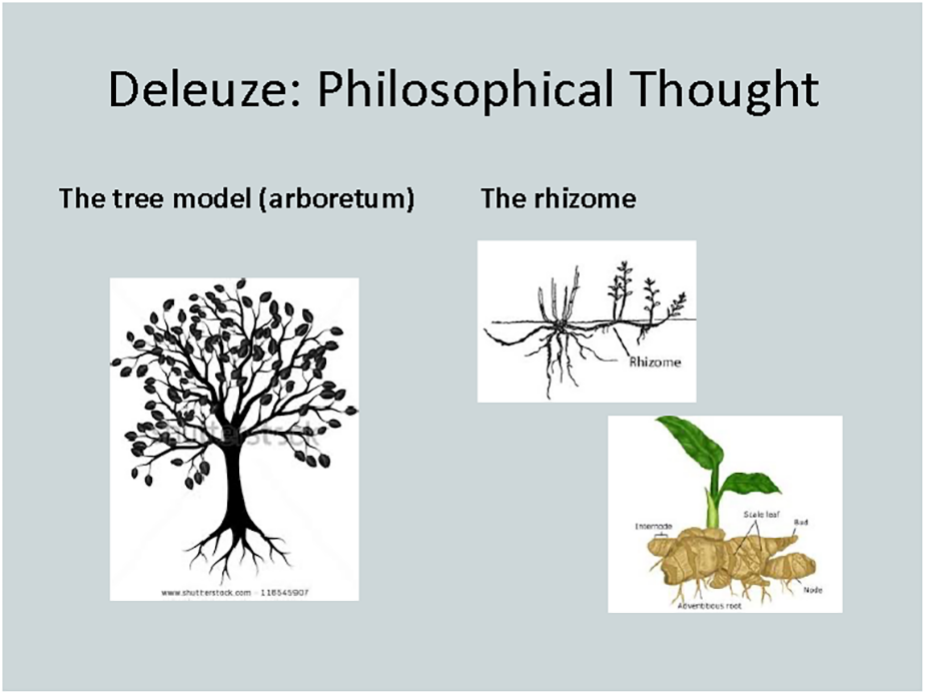
A slide from lecture 'dance performance ethnography', the dancing body in motion.

At this point, I wanted to introduce Deleuze's (49) poststructuralist theory as a way of disrupting “discourse” of the taken-for-granted constructions of the dancing body. We read two Deleuzian-inspired articles on dance (43, 50) to rethink the binary construction of the feminine dancing body to consider it rather as a body “in flux”. We studied Deleuze's (49) imagery to add visuality to our concept of performativity (Figure 3):

What can these mean in performance ethnography work?

Considering that Deleuze's (49) philosophical thought is complex, I have often avoided introducing it at the masters level for the fear of only superficial engagement of his very sophisticated toolkit. While we did not have time to read a substantial amount of Deleuze's work, I tried to make a careful effort to relate his concepts to our previous discussion of social theory. This, I hoped, helped illustrate how Deleuze's theory differs from phenomenology and critical theory and then can add to our work as dance researchers.

Janita was, however, encouraged to think about what a theoretical concept—not necessarily Deleuzian—may mean for her performance ethnography. How did it move her? How did it make her move? How can she draw from her own experiences and intertwine them with the theory/movement mix? Are there images, like Deleuze's (49) “tree” or “rhizome”, or words that can inspire movement? Or can movement inspire words? With this rather conceptual and abstract advice, I sent Janita to create her own performance ethnography draft to be presented a week later in a practice presentation.

Janita:

Why do I dance? Who am I as a dancer—or am I a dancer at all? How does dance make me feel? I have asked those questions multiple times. Dancers are often passionate about dance, but simultaneously, it can make us feel bad about ourselves. One day, we may decide never to return to a studio, but the next day, we find ourselves stretching at the ballet barre again.

The performance ethnography combined the material body and social body, body and mind, and practice and theory into an embodied presentation. I enjoyed this combination, as it was a new and exciting way of representing research. I felt that the verbal presentation supported the dancing, and the movements complimented the texts. Some personal experiences were also more easily presented through the body.

I acknowledged that there was more vulnerability involved in this kind of performance: it was not only about writing a paper or creating a dance performance, both of which alone I had some prior experience in. I still felt great!

I have not done anything like this before: I am publicly talking and dancing, often at the same time, and sharing both the results of my academic work and my personal experiences—being vulnerable on stage in multiple ways.

Pirkko:

I was apprehensive of how Janita may respond to this assignment as I had seen many attempts in the name of performance ethnography that lacked performativity: theoretical depth, connection to a main conceptual premise, or performance ability. I even mentioned to a passing colleague that I was going to assess performance ethnography by a student and was very unsure of the quality of the outcome. The same colleague saw me coming back and asked: So, how did go? Unbelievable! I answered. I was amazed by the insight, creativity, connection to theoretical concepts, and performance quality of using props, voice, and dance that Janita had achieved based on my rather open and vague instructions. This was absolutely remarkable from a graduate student! With only minor “edits”, Janita was now ready for the final performance.

Janita:

I am confident that performance ethnographies are meaningful for the choreographer and researcher. Perhaps these performances may also appear more meaningful for the audience. I am intrigued by Denzin's (41) notion, citing Conquergood (51), of performance “moving” other people. Perhaps by actually moving, we can “move” other people more.

Pirkko:

We had invited several staff members and graduate students to attend Janita's live presentation of her performance ethnography. Many were dancers and I was wondering how they would react to performance ethnography that has a different purpose from a dance performance. The overall reaction was extremely positive!

Janita:

I wondered if combining theory and practice, speaking and moving to represent research, could be used to create dance pieces in different settings. In fact, this course inspired several other performances: I presented a revised version of this performance ethnography at our Faculty's student conference, ReCon, and created another one about my thesis research to be performed in a dance showcase. Pirkko and I also collaborated on a performance for Pirkko's keynote address at the North American Society for the Sociology of Sport conference.

Pirkko:

Later in the same year, I was asked to give a key note address at the North American Society for the Sociology of Sport (the main international sport sociology association) conference. As this was a major honour, I began early to prepare my presentation that was inspired by the potential of new materialism to enrich our sub-field. I presented a practice talk to a number of colleagues and graduate students who gave me plenty of constructive critique including questioning what all my very theoretical discussion can possibly mean in the practice of kinesiology research. I thought of the “Dancing Body in Motion” course and I thought of performance ethnography and how it can embody the performing body and move theoretical thinking. I asked Janita to recreate part of her original 12-minute performance ethnography for my key address. Based on comments from the audience of international scholars, this was the most memorable part of my keynote, the one that exemplified critical material and social scholarship much more evidently than simply writing or talking about it. In a later article based on my keynote, I, inspired by the course and the performance ethnography assignment, conclude that “experimentation with research representation that canvases the analyses of the moving body as both material and social can activate social change by challenging the boundaries of neoliberal knowledge production” (32).

**Pirkko:** When we now watch the recording of your performance ethnography as presented at the end of the course and a revised form in ReCon, what are your thoughts?

**Janita:** Watching it back, it seemed quite raw and explorative, just learning about the combination of text and movement. I don't know where some of those choices came from.

**Pirkko:** My first thought was “Oh my gosh, I made Janita do so much talking!” It seemed very theory-driven.

**Janita:** Very theory-driven! There was more dancing in the second version, the ReCon conference performance. That made me wonder: Why was there less movement in the first performance? Because dancing was definitely the most exciting part of the course for me. I wonder if this being an academic presentation made me feel like there has to be more theory. I spent a lot of time downstage with the papers filled with theory lined up on the floor. I used the space better in the ReCon performance ethnography, and there was more movement combined with the text.

**Pirkko:** Your performance ethnography drew from a range of theoretical concepts, but you concluded with Foucault. Did you plan to use the other theoretical perspectives to arrive at Foucault or how did you arrive at this order?

**Janita:** I wanted to use the theories we had discussed and then end with poststructuralism and Foucault to further problematize the issue of dancer identity since I had learned about Foucault's (52) concept of discipline and disciplinary techniques in a previous course and it was a potential theoretical perspective for me to use in my thesis research.

But looking back, the Foucauldian part could have been more prominent. In the ReCon performance ethnography, I used the space more widely for discussing the theory, but for the Foucauldian part, I was downstage. I emphasized how the spacing made that part of the theory more powerful. In any case, I wonder what is a good relation between movement and text in these performances. Another aspect I now found odd was when I talked about the hamstring tear I was recovering from, yet I was still sitting in a deep hamstring stretch.

**Pirkko:** I didn't mind the hamstring part at all—I thought it represented the text quite well.

**Janita:** That's true. It made the point of the text without saying it or being too literal.

**Pirkko:** Exactly. Movement in performance ethnography does not necessarily need to be representational or gestural.

**Janita:** The theme “dancing for myself” arose from my personal experiences and learning journal, and I discussed it through the theory. In that moment, I truly felt I was dancing for myself more than ever before. I'm realizing now how it was the result of our theoretical discussions that I understood how the dancing body is a social construct. Questioning some of those ideals and expectations helped me feel “freer” in my movement and dancing body. After the final performance ethnography, I wrote in my learning journal: “Today, I feel better about myself as a dancer than I have in a long, long time”.

**Pirkko:** The video recordings also reminded me of the realities of the stages that we perform on. These performances bring the dancing body very close to the audience. In your performance at ReCon, you were so close to the audience that you could almost touch them.

**Janita:** I was just thinking if having the audience focus on the body and movement is good or if it hinders getting the information out there. Since we research the body, it's great to bring it into these presentations and closer to the people. It's not separate from what we talk about. Now I see how new materialist that is. It shows how movement can communicate a lot, and it is powerful when it does so.

## Discussion

In this paper, we have discussed how embodied writing can inform a graduate-level dance course in a Kinesiology Faculty. Our embodied approach aimed to challenge the dualistic approach that assigns physical sciences (anatomy, biomechanics) and the social sciences as separate ways of knowing the dancing body in graduate education. Informed by a new materialism, the “Dancing Body in Motion” course embraced an embodied process of analyzing dance technique, lived experience of the dancing body, social theory, and performance and as such, offered opportunities for holistic knowledge of the dancing body. The course assessment reflected the embodied approach through a reflective journal and a performance ethnography that enabled Janita to include both physical and social lived experiences of dance. The performance ethnography also introduced Janita to an alternative way of research representation. We continued the embodied writing practice in this paper through an embodied representation of our reflections on the course.

In the learning journal, Janita was able to write about her embodied experiences as they were evoked during the course. The performance ethnography, however, offered ways to disrupt the text-based assessment by moving beyond the “diary” (3) to encourage experimentation with alternative ways of research representation at the graduate-level dance education. We both offered a substantial amount of reflection on the performance ethnography that, as one way of embodied writing practice, obviously was the most impactful aspect of the course for us. The performance ethnography also expanded the knowledge (and the course itself) beyond the course through a public presentation for the Faculty as well as several conference presentations. Therefore, as an embodied assessment, it can assist dance educators and students, not only to move beyond textual assessment, but also gain visibility for dance practice as a valuable element of research presentation. As a creative approach to embodied reflection (3), the performance ethnography can act as a meaningful form of assessment for a dance graduate student. As a dancer, Janita also discovered, similar to the students in Bradshaw-Yerby's (1) course, that movement practice—that she felt most comfortable with—aided with her writing process. Consequently, performance ethnography as an assessment can advance both writing and movement skills at graduate-level dance education. In addition, it can act as a personally fulfilling and engaging mode of assessment “that validates the lived or danced experience while helping to communicate that experience comprehensively to a wider audience” (2).

Janita has continued to embrace the opportunity to discuss dance in an embodied manner: its practices, her experiences, and social theory. The embodied writing practices, thus, can prepare graduate students in dance for their future careers more fully than text-based research writing alone (3). Janita, indeed, found that the performance ethnography gave her further confidence to pursue a career as a dance artist. The knowledge gained in the course now also guides Janita's embodied dance teaching: she now helps the students to focus more on how the body feels instead of its looks; she now implements (small) changes in the exercises and movement practices for safer and more meaningful student dance experiences. Janita continues to remind herself of her responsibility as a teacher. The way we teach will have an effect on the dancers: how they learn to move safely; how they learn to sense their bodies; and how they end up feeling about their bodies and, ultimately, about their selves. In addition to teaching, Janita's artistic practice is now informed by the larger picture of dance and the arts in western society. She considers how to create more meaningful, inclusive, and well-rounded (body and mind) art pieces, performances, and spaces as she explores new ways to define how dance and the arts are practiced, presented, and seen. Janita currently characterizes her artistic work as theory-informed: Connecting different knowledge and combining theory and practice helps her consider the “why” behind practices and the sociocultural context(s).

Embodied research writing, like embodied creative practice of dance that “is able to transcend dualist divides between the body and mind”, can “sweep” social science research onwards (53). Embodied research writing on dance, then, can facilitate a reaching across disciplinary boundaries to engage with the new materialist premise of capturing both the matter and its mattering in the complex contemporary world (32). This may mean disrupting “the comfortable, taken-for-granted (striated) academic spaces of reading, thinking, and knowing” (45) to experiment with ways to move beyond mere textual representation to include sensory aspects previously excluded from research writing (53). Dance researchers, with a sensibility to movement experiences, can be forerunners in this type of experimentation that foregrounds the uniqueness of dance as a human bodily endeavor. For Pirkko, the course certainly served as an inspiration to follow a broader scholarly path as a new materialist scholar who continues to experiment with scholarly writing and performance.

## Conclusion

Based on our experiences in the “Dancing Body in Motion” course, we now endorse future research that explores further ways of how embodied writing can be utilized in teaching (teacher perspective), learning (student perspective), and representing research in dance education in different contexts including kinesiology. Our approach to embodied writing drew from a new materialist perspective and was aimed at the graduate level, but some aspects of embodied writing can also be used in other, such as recreational dance, settings to bring in lived experiences as inspiration to choreography and embodied performance. Considering the positive response to performance ethnography as a way of representing research, we are encouraged to use it more in a conference setting as an alternative to the usual written and spoken presentations. Having an opportunity to write about the course—its new materialist premise, the attempts to combine theory and practice, the material body and the social body, and the performance ethnography that brought both of these aspects together—offers another invaluable venue for our embodied writing work in the academic world.

## Author's note

The social theory section and the new materialist, poststructuralist section included journal articles as reading material. The phenomenology discussion was supported by the works of Sheets-Johnstone (12), Fraleigh (47), Aalten (19), and Rouhiainen (16). The discussion on critical (feminist) and cultural studies approaches to the dancing body was supported by McEwen and Young (54), Barr and Oliver (55), Wolff (56), Chatterjea (57), and Boyd (58). The section on new materialism and Deleuzian poststructuralism was supported by Denzin (41), Giardina (59), Fullagar (60), Markula (44), Lock (50), and Portanova (61).

## Data Availability

The original contributions presented in the study are included in the article/Supplementary Material, further inquiries can be directed to the corresponding author.
